# The International Advanced Practice Nurse Integration Model: A Response to Recent Commentaries

**DOI:** 10.34172/ijhpm.9777

**Published:** 2026-02-08

**Authors:** Joshua Porat-Dahlerbruch, Tatyana Miller, Judith Shamian, Moriah Ellen

**Affiliations:** ^1^Department of Acute & Tertiary Care, School of Nursing, University of Pittsburgh, Pittsburgh, PA, USA.; ^2^Israel Implementation Science and Policy Engagement Centre, Ben-Gurion University of the Negev, Be’er Sheva, Israel.; ^3^Herzog Hospital, Jerusalem, Israel.; ^4^International Council of Nurses, Geneva, Switzerland.; ^5^Department of Health Policy and Management, Guilford Glazer Faculty of Business & Faculty of Health Sciences, Ben-Gurion University of the Negev, Be’er Sheva, Israel.; ^6^Institute of Health Policy Management and Evaluation, Dalla Lana School of Public Health, University of Toronto, Toronto, ON, Canada.

## Introduction to Advanced Practice Nurse Integration

 Per the International Council of Nurses (ICN), an advanced practice nurse (APN) is “a generalist or specialized nurse who has acquired, through additional graduate education, the expert knowledge base, complex decision-making skills, and clinical competencies shaped by the context in which they are credentialed to practice.” APNs, particularly when authorized to prescribe, can help enhance access to care by filling provider shortages and augment care quality through a holistic approach.^1^ Robust research on outcomes linked to APN care in countries with long histories of the role (eg, United States and Canada) have demonstrated less acute care utilization, higher patient satisfaction, and prescribing quality similar to physicians.^2-4^

 However, simply introducing APNs into health systems does not translate directly to improved care access and quality.^1^ APN integration mediates the relationship between APN introduction and outcomes.^5^ In other words, to reap the positive outcomes linked to APN care, APNs must be able to function according to their expanded scope and education.^5,6^ There are consistent issues across countries hindering the integration of APNs at all levels of the health system—macro (national, regional), meso (organization), and micro (care team) levels.^6-8^ Examples of macro-level factors include unclear or restrictive scope of practice laws/regulations, reasonable financial support for APN services, and public awareness of APN roles.^9^ Meso-level factors include physical resources (eg, APN access to electronic health records and office space), evaluation of APN integration procedures/policies, and leadership messaging about APNs.^9^ Micro-level factors include interprofessional relationships and patient trust in APN care.^9^ These barriers hindering APN integration lead to human resource and systemwide inefficiencies.^1^

 APN integration hindrances persist internationally in large part because decision-makers lack guidance on developing policies for integrating APNs.^6^ Much of the existing literature focuses on single countries or settings,^6^ though three seminal literature reviews concluded that hindrances to APN integration are common across countries.^6-8^ Further, APN integration can be conceptualized as an implementation science issue—APNs are an evidence-based intervention for improving care quality and access, but policies and practices must support their uptake.^6^ Implementation science adopts the view that broadly applicable models can and should be developed to guide implementation (or here “integration”) across contexts, so long as they are tailorable to specific population needs.^1^ In all, there is a need and appropriate fit to develop a globally applicable model guiding APN integration.

## The International Advanced Practice Nurse Integration Model

 The impetus for this paper is to respond to commentaries on our article reporting the development of policy interventions for integrating APNs in Israel.^10-12^ In her commentary, Grinspun^12^ states, “the taxonomy can evolve into a global resource for strengthening [APN] roles and advancing interprofessional collaboration.” Grinspun’s statement supports our argument that an international model for integrating APNs is not only feasible but also necessary to ensure efficacious APN workforce development worldwide.

 As such, we are utilizing this correspondence to introduce the International APN Integration Model. We developed the International APN Integration Model based on research following our article published in *International Journal of Health Policy and Management (IJHPM)*.^10^ The studies were based on a series of international and single country studies, which we coalesce here into a single location for practical use by policy-makers, managers, and researchers (Figure).^1,5,6,9,10,13,14^

**Figure:**
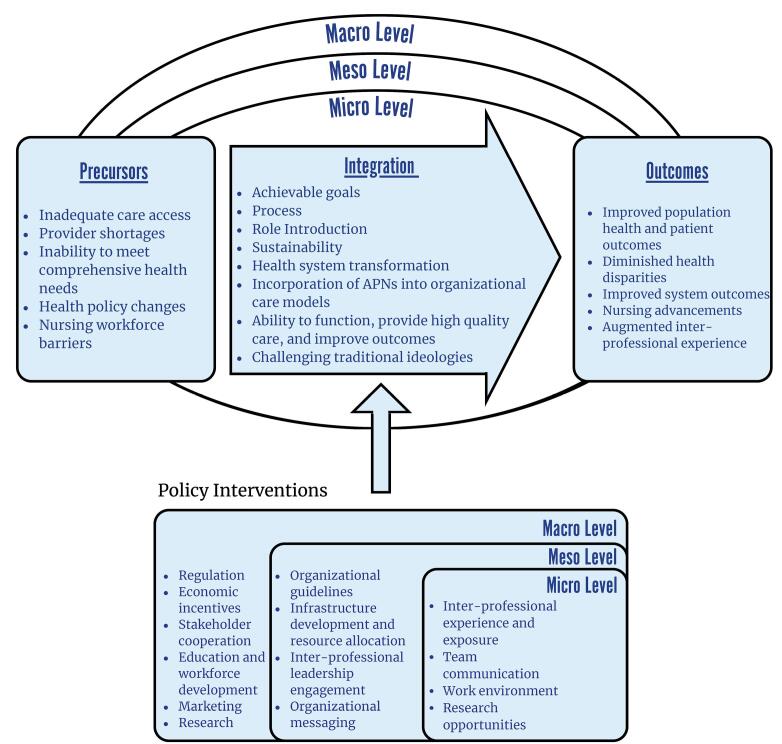
International Advanced Practice Nurse Integration Model. The model describes the process of integrating APNs into a health system. Once APNs are introduced, the process of integrating the role ensues. Facilitators and barriers at macro (nation, jurisdiction, region), meso (care organization), and micro (care team) levels affect integration progress. To magnify and diminish the impact of facilitators and barriers, respectively, policy interventions should be implemented. Once integration progress reaches a point at which APNs are functioning at or close to the full extent of their education and scope of practice, desired outcomes (eg, improved care quality and access) can be achieved. The model is adapted from our prior publications.^5,13^ Abbreviation: APNs, advanced practice nurses.

 Following the flow of the APN integration process, the precursors indicate the impetus for introducing APNs into a health system.^5,6^ Once introduced, the integration process begins.^6^ When reaching advanced stages of integration, APNs contribute to care outcomes.^6^ Further, the integration process spans macro, meso, and micro levels—that is, factors at all three health system levels affect the integration process.^9^ Policy interventions are implemented during the integration process and may target any three of the health system levels.^13^ Precursors and outcomes may lay at a specific level—eg, a need and expectation to enhance care access across a nation (macro), to augment the provider workforce in a specific care organization (meso), or to improve patient-centered, holistic care in a specific clinic (micro).^6^ Figure includes overarching categories of APN integration precursors, attributes, outcomes, and policy interventions—more detailed descriptions can be found in Supplementary files 1 and 2.

## The Paradox of Advanced Practice Nurse Integration as an Impetus for the Model

 In their commentary, Shadmi and Lopez^11^ describe the importance of coordinating APN integration efforts with academic institutions, which raises a paradox faced by policy-makers. By the ICN’s definition of APNs, they are educated at the graduate level. Graduate-level education requires a cadre of doctoral or master’s prepared nurses to educate the first generation of APNs within the country’s population and system context. As such, in countries without existing graduate-level nursing education infrastructure, how can APNs be educated to meet the ICN’s definition of an APN? Countries newly developing the APN role tend to struggle with this notion.^14^ Indeed, there have been examples of international partnerships in which faculty from countries with greater academic nursing infrastructure partner to educate the first generation of APNs in a particular specialty.^15^ These international partnerships, nevertheless, are logistically difficult to establish and tend to produce only a few APNs, not a number sufficient to launch the profession.

 In Israel, the solution to this paradox was to require a bachelor’s and master’s degree (one of which must be in nursing—typically the bachelor’s degree) for admission to a post-master’s APN certificate program. This approach indeed fits the ICN’s definition of APN. Research in Israel shows that most stakeholders (APNs, managers, and policy-makers) do not believe that the quality of the program is based on the type of degree/certificate conferred.^10,14^ However, experts have debated whether the APN program should lead to master’s degree conferral.^11,14^

 Education is not the only APN integration paradox. Our research in both Israel and across 10 countries with existing APN roles has shown that physician resistance to APNs is often solvable with exposure to working with APNs.^10,13^ However, physicians must be open to working with APNs to gain exposure. Physicians in Israel often worked with APNs because managers unilaterally inserted them into the care team or because they already worked with APNs during residency in other countries.^10^

 These are just two examples of paradoxes with which policy-makers and managers struggle when developing APN integration policies. These paradoxes reinforce the need to promote an evidence-based model to guide policy-makers and managers in tackling APN integration policies.

## Moving Toward International Adoption of the Advanced Practice Nurse Integration Model

 The original paper, together with the rich commentaries by Shadmi and Lopez and Grinspun, demonstrate that the APN Integration Model provides clear and actionable direction for advancing comprehensive integration to strengthen health systems and population health.^10-12^ The central challenge now lies in translating this accumulated knowledge into global, regional, national, and local practice. Achieving this requires engagement from organizations with the policy authority, financial capacity, and accountability mechanisms necessary to enable full APN integration. Global institutions (eg, World Health Organization (WHO) and its regional offices, World Bank, and regional governance bodies like the European Union and Southern African Development Community) are uniquely positioned to operationalize the APN Integration Model at an international scale. By embedding APN integration within workforce planning, financing, regulation, and performance accountability frameworks, these organizations can support the development of a strong, resilient, and economically viable health workforce, while accelerating progress toward Universal Health Coverage and the Sustainable Development Goals 2030 agenda.

## Conclusion

 The commentaries on our Israel-based study^10^ focus on developing policy interventions to improve APN integration and indicated the need for an international version of the APN Integration Model.^11,12^ In this commentary, we coalesce our prior work to present a unified International APN Integration Model. Moving forward, we plan to publish measurement tools for evaluating APN integration progress and the pre-post impact of interventions aimed at improving integration.

## Acknowledgements

 The content in this article is adapted from our prior publications on APN integration. Appropriate permissions were obtained to include any previously published content. We would like to thank Ivy Chen for preparing the figure and supplementary files.

## Disclosure of artificial intelligence (AI) use

 Not applicable.

## Ethical issues

 Not applicable.

## Conflicts of interest

 Authors declare that they have no conflicts of interest.

## Supplementary files


Supplementary file 1. Precursors, Outcomes, and Attributes of Advanced Practice Nurse Integration.

Supplementary file 2. Macro-, Meso-, and Mico-Level Policy Interventions for Improving Advanced Practice Nurse Integration.
