# Supplementary material for: The International Advanced Practice Nurse Integration Model: A Response to Recent Commentaries
Source: Int J Health Policy Manag. 2026 Feb 8;15:9777. doi: 10.34172/ijhpm.9777 (PMC13034194; doi:10.34172/ijhpm.9777)
Supplement: Supplementary file 1 — Precursors, Outcomes, and Attributes of Advanced Practice Nurse Integration. [file ijhpm-15-9777-s001.pdf]

SUPPLEMENTARY FILE 1: Precursors, Outcomes, and Attributes of Advanced Practice Nurse Integration

Article Title: The International Advanced Practice Nurse Integration Model
Journal Name: *International Journal of Health Policy and Management*

Author Information:
Joshua Porat-Dahlerbruch[1,2] (Corresponding Author) (JOP186@pitt.edu)
Tatyana Miller[3]
Judith Shamian[2,4]
Moriah Ellen[2,5,6]

[1] Department of Acute & Tertiary Care, School of Nursing, University of Pittsburgh, Pittsburgh, Pennsylvania, United States

[2] Israel Implementation Science and Policy Engagement Centre, Ben-Gurion University of the Negev, Be'er Sheva, Israel

[3] Herzog Hospital, Jerusalem, Israel

[4] International Council of Nurses, Geneva, Switzerland

[5] Department of Health Policy and Management, Guilford Glazer Faculty of Business & Faculty of Health Sciences, Ben-Gurion University of the Negev, Be'er Sheva, Israel

[6] Institute of Health Policy Management and Evaluation, Dalla Lana School of Public Health, University of Toronto, Toronto, Ontario, Canada

Note: This file's content is adapted from the following reference

Porat-Dahlerbruch J, Chen I, Cahir K, Blumenthal NP. Adaptation of the Nurse Practitioner Integration Model for older adult primary care models. Geriatric Nursing. 2025;65(5):1-8. doi:10.1016/j.gerinurse.2025.103453

Precursors	Descriptions
Inadequate care access	Patients often experience gaps in coverage or long wait times to access care. This includes the time it takes to schedule a face-to-face appointment and the wait time at the care facility. Inadequate access to care is common, particularly in primary care clinics and emergency departments.
Provider shortages	There is a shortage of care providers and an unequal distribution of them to meet the population's care needs. Both the provider workforce and the population are aging, which can drive shortages among the entire workforce or specific specialties.
Inability to meet comprehensive health needs	The supply of care services and expertise lag the increased demand for patient and population health needs across the care continuum.
Health policy changes	National policy changes impact care organizations' ability to meet care demands at the local level. Policy changes can include changes in work-hour requirements, efforts to improve patient outcomes (e.g., decreasing length of stay and readmissions), and changes in public and/or private health insurance coverage.
Nursing workforce issues	Efforts to improve nursing job outcomes include greater autonomy, a personalized role identity, reduced burnout, enhanced well-being, and better application of knowledge and expertise in practice.

Outcomes	Descriptions
Improved population health and patient outcomes	Indicators of high value and cost-effective patient and population outcomes associated with APN care. Examples include decreased length of stay, decreased complication rates, decreased rates of morbidity, decreased risk of mortality, improved care continuity, improved patient safety, more patient contact hours, and higher patient satisfaction.
Diminished health disparities	Increased ability for a health system to supply high-quality care options tailored to underserved and under-represented patients and populations.
Improved system outcomes	Indicators of improved health system efficiency and quality (e.g., reduced costs, shorter wait times for an appointment, provider shortage alleviation, improved care access, and decreased unnecessary hospital and emergency department use).
Nursing professional advancement	Development of the nursing workforce to advance education opportunities, advocate for policy changes, and enhance the visibility of nurses in the healthcare team and society.
Augmented inter-professional experience	Indicators of improved interprofessional teamwork and collaboration for all members of the healthcare team (e.g., improved job satisfaction, decreased provider workload, increased provider retention, improved resource utilization, improved clinician well-being, and improved evidence-based care delivery).

Attributes	Descriptions
Achievable goal	APN integration was described as having an end goal of full or complete incorporation into care models. Once nearing the achievement of full APN integration, APNs can begin to improve care quality and access significantly for the population which they service.
Process	APN integration occurs over a span of time as opposed to an instance. The process was described as iterative and requiring change, inspiration, acceptance, and intervention. Throughout the process, there is change, role development, policy revision, acceptance, and overcoming challenges. The length of the APN integration process is affected by certain factors (i.e., facilitators and barriers).
Role introduction	APN integration is initiated by the introduction of the APN role. Effective role introduction often involves organizational and/or government communication of the APN role so that the healthcare workforce and patients are aware of APN presence and abilities distinct from physicians, physician assistants, and non-prescribing nurses. Moreover, role introduction involves policymaking to expand the care provider workforce by establishing the APN role.
Sustainability	The integration of APN requires a change in the care model structure to accommodate and incorporate APNs (i.e., legislative changes permitting licensed APNs to provide and prescribe care). In more developed care models, legislative and/or regulatory action from government healthcare authorities are often required to guide organizations, APNs, and healthcare professionals on APN scope of practice.
Health system transformation	APN integration may lead to health system transformation. Where integration is more advanced, APNs are utilized increasingly in primary care with physicians more focused on acute care. APNs, thus, may cause a shift in care specialization and health system structures.
Incorporation of APNs into organizational care models	APN integration involves the addition and inclusion of APNs in healthcare teams to a point at which APNs, providers, and other team members view APNs as care partners. When successfully incorporated, APNs can function autonomously within the bounds of their scope. When APNs are incorporated into care models, it becomes clear to the care team that APN practice is grounded in nursing practice, which creates a unique niche within care teams.
Ability to function, provide high care quality, and improve outcomes	This attribute is the capacity for APNs to improve patient outcomes and care quality. APNs cannot maximally contribute to care without incorporation into organizational care models. Moreover, this attribute is linked to achievable goal as much of the purpose for APN integration is to improve care and system outcomes.
Challenging traditional ideologies	APN integration involves interprofessional, intra-professional, and societal rethinking of nursing roles to accommodate expansion of the practice scope. Within care model hierarchies, nurses are often viewed as non-prescribing clinicians who, in addition to nursing care, carry out physician orders. APNs introduce alterations to this conventional ideology. The public and healthcare professionals must adjust their view of nurses to accommodate an expanded scope.
