# Supplementary material for: The International Advanced Practice Nurse Integration Model: A Response to Recent Commentaries
Source: Int J Health Policy Manag. 2026 Feb 8;15:9777. doi: 10.34172/ijhpm.9777 (PMC13034194; doi:10.34172/ijhpm.9777)
Supplement: Supplementary file 2 — Macro-, Meso-, and Mico-Level Policy Interventions for Improving Advanced Practice Nurse Integration. [file ijhpm-15-9777-s002.pdf]

SUPPLEMENTARY FILE 2: Maro-, Meso-, and Micro-Level Policy Interventions for Improving Advanced Practice Nurse Integration

Article Title: The International Advanced Practice Nurse Integration Model
Journal Name: *International Journal of Health Policy and Management*

Author Information:
Joshua Porat-Dahlerbruch[1,2] (Corresponding Author) (JOP186@pitt.edu)
Tatyana Miller[3]
Judith Shamian[2,4]
Moriah Ellen[2,5,6]

[1] Department of Acute & Tertiary Care, School of Nursing, University of Pittsburgh, Pittsburgh, Pennsylvania, United States

[2] Israel Implementation Science and Policy Engagement Centre, Ben-Gurion University of the Negev, Be'er Sheva, Israel

[3] Herzog Hospital, Jerusalem, Israel

[4] International Council of Nurses, Geneva, Switzerland

[5] Department of Health Policy and Management, Guilford Glazer Faculty of Business & Faculty of Health Sciences, Ben-Gurion University of the Negev, Be'er Sheva, Israel

[6] Institute of Health Policy Management and Evaluation, Dalla Lana School of Public Health, University of Toronto, Toronto, Ontario, Canada

Note: This file's content is adapted from the following reference

Porat-Dahlerbruch J, Miller T, Boyd J, Ellen ME, Clark RRS. The International Advanced Practice Nurse Integration Policy Intervention Taxonomy: A 10-Country Nominal Group Consensus Technique Study. Int Nurs Rev. 2025;72(3):1-10. doi:10.1111/inr.70096

Table S2.1: Macro-Level Policy Categories and Interventions

Policy Category	Interventions
Regulation	• Clearly define the APN role across systems.<br>• Define APN scope of practice that includes autonomous practice.<br>• Differentiate the APN discipline.
Economic Incentives	• Reimbursement and salaries for APN care at a professional rate.<br>• Provide economic incentives for organizations to recruit and retain APNs and promote positive work environment.<br>• Subsidize costs of sending nurses for APN education (e.g., time off payments).<br>• Incentivize organizations for APN contributions to the system and patient care.
Stakeholder Cooperation	• Coordinate APN integration efforts with national interprofessional groups.<br>• Promoting APN self-regulation on parity with other professions.<br>• Work with health professional, hospital, and care organization stakeholder groups when creating a national plan for integrating APNs.<br>• Communicate APN scope of practice to relevant stakeholders and partners.
Education and Workforce Development	• Align and monitor the number of APNs educated in accordance with workforce position availability and population needs across specialties.<br>• Create and fund more positions for APNs.<br>• Transition into standardized competency-based education.<br>• Fund transition to practice programs.<br>• Incentivize preceptorship programs.<br>• Subsidize universities to open APN degree programs.<br>• Establish APN program curricula based on existing international evidence.<br>• Train faculty with relevant education according to the International Council of Nurses (2024) Global Strategic Plan.
Marketing	• Initiate an advertising campaign to inform the public of the new APN role.<br>• Begin an information campaign on the APN role targeted at health professional colleagues.<br>• Expose high school and college students to the APN role and career mobility.<br>• Differentiating APNs from other healthcare services in marketing campaigns.
Research	• Establish international cohesion through research so that there can be a gold standard understanding of the APN role.<br>• Conduct research on APN-linked health system and patient outcomes (access utilization quality and cost).<br>• Evaluate the current utilization of APNs across the systems and sectors including health and academic settings.<br>• Synergize research efforts between international, national and regional levels.

Table S2.1 Caption: The macro level refers to the national, jurisdictional, regional, or international level. APN=advanced practice nurse

Table S2.2: Meso-Level Policy Categories and Interventions

Policy Category	Interventions
Organizational Guidelines	• Clearly define the APN role in both clinical and leadership domains.<br>• Clearly describe APN scope of practice within the organization.<br>• Create an organizational care model that includes APNs and recognizes full scope of APN care.<br>• Promote equitable APN involvement in leadership, shared decision making and policy making.
Infrastructure Development & Resource Allocation	• Outline a formal plan for APN integration.<br>• Integrate APNs across all settings.<br>• Consider establishing APN leadership and reporting structures.<br>• Provide adequate support staff and resources to APNs.<br>• Establish a leadership group for constant policy development for the APN role with representation from all organizational stakeholder groups.<br>• Clearly define APN pay schedules and salary structures in parity with other health care professionals.<br>• Systematically assess APN integration progress, communicate findings internally & update policies accordingly.<br>• Assure data and technology infrastructure to APNs that promotes full access.<br>• Develop onboarding programs and ongoing support for APNs.<br>• Include role title, degrees, & certifications after APN names.<br>• Create data infrastructure clearly attributing APN services to patient and system outcomes.<br>• Encourage evidence-based practices to promote recruitment and retention.
Interprofessional Leadership Engagement	• Host interprofessional workshops and seminars with all providers.<br>• Promote interprofessional decision making.<br>• Promote interprofessional educational experiences.
Organizational Messaging	• Communicate the APN role, education/training, and scope of practice to clinicians throughout the integration process.<br>• Disseminate messaging supportive of APNs to the organization's employees.<br>• Allocate time to educate organizational leaders on APN care benefits.
Research	• Establish international cohesion through research so that there can be a gold standard understanding of the APN role.<br>• Conduct research on APN-linked health system and patient outcomes (access utilization quality and cost).<br>• Evaluate the current utilization of APNs across the systems and sectors including health and academic settings.<br>• Synergize research efforts between international, national and regional levels.

Table S2.2 Caption: The meso level refers to the care organization level, such as a hospital, hospital system, or healthcare maintenance organization. APN=advanced practice nurse.

Table S2.3: Micro-Level Policy Categories and Interventions

Policy Category	Interventions
Interprofessional Experience & Exposure	• Demonstrate APN holistic care and value to the care team.<br>• Assure infrastructure that health care team members are aware of APN value and holistic care methods.
Team Communication	• Institute regular care team meetings to promote consistent communication.<br>• Educate care teams on interprofessional communication skills.
Work Environment	• Encourage appropriate mentoring relationships within the care team.<br>• Provide administrative support including infrastructure, resources and professional skill development.<br>• Ensure that patients and families are aware of the APN role.
Research Opportunities	• Promote APN-led quality improvement research.<br>• Encourage implementation of evidence-based practice.<br>• Advocate for constant evaluation of current clinical practices.

Table S2.3 Caption: The micro level refers to the care team in a clinic or unit at the point-of-care.
